# Supplementary material for: Were ancient foxes far more carnivorous than recent ones?—Carnassial morphological evidence
Source: PLoS One. 2020 Jan 10;15(1):e0227001. doi: 10.1371/journal.pone.0227001 (PMC6953794; doi:10.1371/journal.pone.0227001)
Supplement: S1 Table — (DOC) [file pone.0227001.s001.doc]

**S1 Table**

List of the fossil first lower molars of the arctic fox (*Vulpes lagopus*) from RBINS, Brussels, used in the study with information about site excavation and left/right position of the molar in the tooth-row
