# Supplementary material for: Were ancient foxes far more carnivorous than recent ones?—Carnassial morphological evidence
Source: PLoS One. 2020 Jan 10;15(1):e0227001. doi: 10.1371/journal.pone.0227001 (PMC6953794; doi:10.1371/journal.pone.0227001)
Supplement: S3 Table — (DOC) [file pone.0227001.s003.doc]

**S3 Table**

AMS dates and their calibration (<https://c14.arch.ox.ac.uk/oxcal/OxCal.html>) of mammals present at the Belgian sites analyzed in this study (anthrop.: anthropological)

| site/sample | species | AMS date | cal BP (95%) | | anthrop. traces | reference |
| --- | --- | --- | --- | --- | --- | --- |
| Trou de Chaleux | | | | | | |
| OxA-V-2216-44 | horse | 12375±50 | 14750 | 14130 | yes | Stevens et al. 2009 |
| OxA-V-2216-45 | horse | 12630±55 | 15220 | 14730 | yes | Stevens et al. 2009 |
| OxA-3632 | horse | 12790±100 | 15640 | 14900 | yes | Charles 1998 |
| OxA-4192 | muskox | 12860±140 | 15840 | 14900 | yes | Charles 1998 |
| OxA-3633 | horse | 12880±100 | 15730 | 15110 | yes | Charles 1998 |
| Goyet third cave Bed 1 | | | | | | |
| Utc-8957 | horse | 12560±50 | 15140 | 14530 | yes | Germonpré 2001 |
| GrA-3238 | muskox | 12620±90 | 15280 | 14460 | yes | Germonpré 1997 |
| GrA-3237 | horse | 12770±90 | 15590 | 14900 | yes | Germonpré 1997 |
| OxA-12121 | muskox | 12775±50 | 15420 | 15060 | yes | Stuart and Lister 2012 |
| OxA-V-2223-48 | horse | 12775±55 | 15440 | 15040 | yes | Stevens et al. 2009 |
| KIA-25296 | large canid | 13680±60 | 16790 | 16270 |  | Germonpré et al. 2009 |
| OxA-6592 | woolly rhinoceros | 16320±140 | 20070 | 19340 |  | Stevens et al. 2009 |
| OxA-11291 | woolly rhinoceros | 23560±230 | 28130 | 27350 | yes | Stuart and Lister 2012 |
| OxA-11292 | mammoth | 23940±180 | 28430 | 27680 |  | Barnes et al. 2007 |
| GrA-3239 | cave hyena | 27230±260 | 31520 | 30850 |  | Germonpré 1997 |
| OxA-12120 | woolly rhinoceros | 29330±160 | 33990 | 33150 |  | Stuart and Lister 2012 |
| OxA-V-2223-44 | horse | 31750±200 | 36130 | 35150 | yes | Stevens et al. 2009 |
| OxA-11294 | mammoth | 32280±280 | 36900 | 35530 |  | Palkopoulou et al. 2013 |
| OxA-11293 | mammoth | 32840±340 | 38120 | 36120 |  |  |
| UtC 8958 | cave hyena | 35000±600 | 40990 | 38430 |  | Germonpré 2001 |
| OxA-20997 | mammoth | 35650±600 | 41510 | 39000 |  | Palkopoulou et al. 2013 |
| GrA-9605 | cave bear | 8770±1180-103 | 44990 | 41360 |  | Germonpré and Sablin 2001 |
| Goyet third cave Bed 2 | | | | | | |
| KIA-22275 | polar fox | 12380±60 | 14800 | 14120 |  | Dalén et al. 2007 |
| OxA-12121 | muskox | 12775±50 | 15420 | 15060 | yes | Hämäläinen and Germonpré 2007 |
| OxA-11767 | giant deer | 23840±260 | 28490 | 27540 |  | Stuart et al. 2004 |
| OxA-21117 | mammoth | 29030±400 | 33970 | 32000 |  | Palkopoulou et al. 2013 |
| OxA-V-2223-49 | horse | 29420±170 | 33970 | 33260 |  | Stevens et al. 2009 |
| GrA-43325 | cave bear | 29800±150 | 34200 | 33650 |  |  |
| OxA-11784 | giant deer | 31590±200 | 35990 | 34990 |  | Stuart et al. 2004 |
| KIA-16289 | cave bear | 34920±330-320 | 40210 | 38710 |  | Hämäläinen and Germonpré 2007 |
| Trou des Nutons | | | | | | |
| OxA-4195 | horse | 12630±140 | 15400 | 14240 | yes | Charles 1998 |
| KIA-22283 | red fox | 15510±70 | 18910 | 18610 |  |  |
| KIA-25298 | wolf | 21810±70 | 26230 | 24840 |  | Germonpré et al. 2009 |
| Trou du Frontal | | | | | | |
| OxA-4197 | horse | 12800±130 | 15730 | 14780 | yes | Charles 1998 |
| Trou Magrite | | | | | | |
| OxA-20055 |  | 41199±1000 | 46640 | 42970 |  | Palkopoulou et al. 2013 |

**References to S3 Appendix**

Barnes I, Shapiro B, Lister A, Kuznetsova T, Sher A, Guthrie D, Thomas MG. Genetic Structure and Extinction of the Woolly Mammoth, *Mammuthus primigenius*. Curr Biol. 2007;17(12):1072-5. DOI: 10.1016/j.cub.2007.05.035

Charles R. Late Magdalenian chronology and faunal exploitation in the North-Western Ardennes. BAR International Series 1998;737:1-246.

Dalén L, Nyström V, Valdiosera C, Germonpré M, Sablin M, Turner E, Angerbjörn A, Arsuaga JL, Götherström A. Ancient DNA reveals lack of postglacial habitat tracking in the arctic fox. PNAS 2007;104:6726-6729.

Germonpré M. The Magdalenian upper horizon of Goyet and the late Upper Palaeolithic recolonisation of the Belgian Ardennes. Bull Inst R Sci N B-S. 1997;67:167-182.

Germonpré M. A reconstruction of the spatial distribution of the faunal remains from Goyet, Belgium. Notae praehistoricae. 2001;21:57-65.

Germonpré M, Sablin MV. Systematics and osteometry of Late Glacial foxes from Belgium. Bull Inst R Sci N B-S. 2004;74:75-188.

Germonpré M, Sablin MV, Stevens RE, Hedges REM, Hofreiter M, Stiller M, Després V. Fossil dogs and wolves from Paleolithic sites in Belgium, the Ukraine and Russia: osteometry, ancient DNA and stable isotopes. J Archaeol Sci. 2009;36:473-490.

Hämäiläinen R, Gemonpré M. Fossil Bear Bones in the Belgian Upper Paleolithic: The possibillity of a Proto Bear-Ceremonialism. Arctic Anthropol. 2007;44:1-30.

Palkopoulou E, Dalén L, Lister AM, Vartanyan S, Sablin M, Sher A, Nyström Edmark V, Brandström MD, Germonpré M, Barnes I, Thomas JA. Holarctic genetic structure and range dynamics in the woolly mammoth. Proc Biol Sci. 2013;280(1770):20131910. doi: 10.1098/rspb.2013.1910

## Stevens RE, Germonpré M, Petrie CA, O'Connell TA. ***Palaeoenvironmental and chronological investigations of the Magdalenian sites of Goyet Cave and Trou de Chaleux (Belgium), via stable isotope and radiocarbon analyses of horse skeletal remains.*** [***J Archaeol Sci***](https://www.sciencedirect.com/science/journal/03054403). 2009;36:653-662. ***doi: 10.1016/j.jas.2008.10.008***

Stuart AJ, Lister AM. Extinction chronology of the woolly rhinoceros *Coelodonta antiquitatis* in the context of late Quaternary megafaunal extinctions in northern Eurasia. Quat Sci Rev. 2012;51:1-17. doi: 10.1016/j.quascirev.2012.06.007.

# Stuart AJ, Kosintsev PA, Higham TFG, Lister AM. Pleistocene to Holocene extinction dynamics in giant deer and woolly mammoth. Nature. 2004;431:684-689
