# Supplementary material for: Were ancient foxes far more carnivorous than recent ones?—Carnassial morphological evidence
Source: PLoS One. 2020 Jan 10;15(1):e0227001. doi: 10.1371/journal.pone.0227001 (PMC6953794; doi:10.1371/journal.pone.0227001)
Supplement: S4 Table — (DOC) [file pone.0227001.s004.doc]

**S4 Table**

List of dental morphotypes used to assess of dental polymorphism in the red fox (*Vulpes* *vulpes*) and the arctic fox (*Vulpes* *lagopus*)

| **group of morphotypes** | **morphotype** | **definition of morphotype** |
| --- | --- | --- |
| **group K**  describes the variation in shape of the posterolingual part of the crown of M_1_ | **K1** | no traces of the conule presence in the concavity between the metaconid and entoconid on the lingual side of the crown |
|  | **K2** | a small singular conule (entoconulid) is present in the concavity between the metaconid and entoconid on the lingual side of the crown |
|  | **K3** | two small conules (postmetaconulid and entoconulid) are present in the concavity between the metaconid and entoconid on the lingual side of the crown |
| **group L**  describes the variation in the shape of the posterobuccal part of the crown of M_1_ | L1 | no traces of the conule present in the concavity between the protoconid and hypoconid on the buccal side of the crown |
|  | **L2** | a small singular conule is present in the concavity between the protoconid and hypoconid on the buccal side of the crown |
|  | **L3** | two small conules are present in the concavity between the protoconid and hypoconid on the buccal side of the crown |
| **group P**  describes variability in development and shaping of central cristid and other crests on occlusal surface of the talonid basin on M_1_ | **P1** | no traces of enamel cristids in central part of talonid basin on M_1_ (variant P-) |
|  | **P2** | variants with short fragments of enamel cristids in central part of talonid basin (variants Pa, Pb, Pab, Pi, Pl) |
|  | **P3** | enamel cristids create partial but not complete join between entoconid and hypoconid (variants Pc, Pcb, Pg - cristid run from hypoconid and it is broken in the central part talonid basin, variants Pd, Pe, Pf, Pid, Pie, Paf, Pad, Phi, Pld, Plf, Ple - cristid runs from the entoconid, and it is broken in central part talonid basin) |
|  | **P4** | variants with complete or almost complete join between entoconid and hypoconid (variants Phd, Phf - almost complete connection between entoconid and hypoconid, variants Pgf, Pgd, Pcf, Pcd, Pcid, Pgid, Pce - complete connection between entoconid and hypoconid) |
|  | **P5** | all variants, in which apart from feature of changeable cristid presence, another enamel ridge on the hypoconid is visible; this ridge originates from the mesial part of hypoconid base and runs in direction of the cusp top medially in relation to cristid oblique (variants Pdj, Pgfj, Padj, Pabk, Padk, Pk, Pkd, Pek, Pfk, Pldk, Pcfk) |
| **group R**  describes the variation in shape of the posterobuccal part of the crown of M_1_ | **R1** | no traces of a hypocingulid presence in the posterobuccal part of the crown of M_1_ |
|  | **R2** | a small hypocingulid present in the posterobuccal part of the crown of M_1_ |
| **group S**  describes the variation in shape of the posterolingual part of the crown of M_1_ | **S1** | no traces of conule presence in the posterolingual part of the crown of M_1_ behind the entoconid |
|  | **S2** | a small conule is present in the posterolingual part of the crown of M_1_ behind the entoconid |
