# Supplementary material for: Were ancient foxes far more carnivorous than recent ones?—Carnassial morphological evidence
Source: PLoS One. 2020 Jan 10;15(1):e0227001. doi: 10.1371/journal.pone.0227001 (PMC6953794; doi:10.1371/journal.pone.0227001)
Supplement: Attachment — Submitted filename: Response to Reviewers.doc [file pone.0227001.s005.doc]

**'Response to Reviewers'**

**My answers to reviewer comments:**

Reviewer #1:

1) If possible, please list institutional catalog numbers for all specimens used in this study as supplementary data. *fixed*

2) Lines 340, 342, and 388: Remove "n. sp." as this suggests that you name the species in this manuscript, which is not the case. *fixed*

Reviewer #2:

In this manusript, Szuma and co-author analyzes a large dataset of extant and fossil arctic and red fox carnassial specimens, using morphotype analysis and a carnivory index to understand morphological changes in fox populations during the Quaternary. This investigation thoroughly examines attributes of carnassial dentition size and morphology in fossil and extant specimens of two species (Vulpes lagopus and Vulpes vulpes) from multiple populations. The authors provide valuable data and intriguing inferences regarding ecological and evolutionary changes in these foxes. The analyses are based on a solid foundation of previous work by the first author and others, and provides an interesting perspective on dental evolution in an ecologically flexible lineage of carnivorans. I find the general topic and data to be worthy of publication, but there are issues with the presentation of statistical findings and organization of the discussion section that need additional work. Therefore, I am recommending major revisions to the manuscript before acceptance. Specific comments are included below:

Abstract:The title of the paper poses a specific question about evolutionary trends in the carnassial morphology of Vulpes, but neither the abstract nor the conclusions specifically answer this question. I suggest revision of both sections to more clearly present the implications of the findings from the analyses.

*The manuscript was supplemented by conclusions about evolutionary trends in the carnassial morphology of Vulpes in discussion and separate last chapter "Conclusions"*

P3L39: recommend "specialized" rather than "special".	*fixed*

P3L41: the bite force / mechanical advantage is maximized in the most posterior teeth. It is more accurate to say that the carnassial tooth is in a position that simultaneously maximizes bite force AND gape. *fixed*

P3L42: "is intimately associated with" rather than "accurately reflects", or include reference showing that carnassial actually accurately reflects diet. *fixed*

Line 50 & Line 100: Please change “Up till” to “Until”

*in these phrase 'up till' is much appropriate*

P5L107: "less carnivorous" would suffice, without using "specialised". *fixed*

P5L108: "to present" rather than "to nowadays". *fixed*

P6L118-119: Incorporating left and right teeth from the same individuals introduces statistical non-independence in the fossil samples. The authors should provide an explanation of how this method choice could affect study outcomes.

*Each fossil first lower molar or part of mandible with the lower carnassial were identified as a separate specimen with own collection number (see Appendix 3, 4). It was impossible to these data found left and right molars of the same individual. Probably many of the teeth came from different individuals. Generally morphotype characters indicate left-right symmetry, also morphotypes groups P, R, S and K, L. However in some specimens we observed unilateral expression of morphotype characters. Determination of the influence of the method impact on the last study outcomes is very difficult in these circumstances. However the number of the ancient samples of the red fox (n=35) and the arctic fox (n=45) seems to be high enough to minimize the method affect.*

Line 176: Please replace “genders” with “sexes.” The word gender is often interpreted as meaning individuals’ concepts of themselves (in the human societal context), whereas the word sex typically refers to the biological differences of males and females. *fixed*

Line 181-182: For improved clarity, I suggest writing “length of M1 (LM1) and width of M1 (WM1)”. *fixed*

P11L252: how could the p value be simultaneously smaller than 0.001 and larger than 0.05? Please clarify. *fixed*

P12L279: A p>0.05 does not imply that the tested attributes "did not vary"; it simple means that samples with differences as large as those observed are expected by chance at higher percentages than the 0.05 level evaluated. Please clarify. *fixed*

Lines 458-468: The concluding paragraph could be enhanced by adding 1-3 sentences summarizing the main findings regarding the arctic fox (Vulpes lagopus), given that the overall focus of the paper has the broader scope of ancient foxes.

*This part of discussion was extended and new citations were included.*

Discussion section in general: lots of results are presented in the discussion section, but I would like to see more discussion of their implications. P18L385 specifically spells out the implications of the dental morphotype analyses in arctic foxes: "a progressive reduction in tooth size and an undermining or predatory specialization in the dental system"; I would like to see similarly clear statements about the red fox data. In addition, the discussion section lacks a summarizing paragraph to integrate the overall conclusions about the question posed in the title: were ancient foxes far more carnivorous than recent ones? Related to this point, the current manusript ends with a section on the "phylogeny of the arctic and red foxes", which is not the focus of this study (authors refer to other research, comparing them to their phenograms, but the data from this specific study does not resolve phylogenetic relationships because no phylogenetic analysis was conducted). Therefore, I recommend a reorganization of the discussion section to bring the focus of the paper back on track.

*fixed*
